# Optimizing Hot Electron Harvesting at Planar Metal–Semiconductor Interfaces with Titanium Oxynitride Thin Films

**DOI:** 10.1021/acsami.3c02812

**Published:** 2023-06-12

**Authors:** Brock Doiron, Yi Li, Ryan Bower, Andrei Mihai, Stefano Dal Forno, Sarah Fearn, Ludwig Hüttenhofer, Emiliano Cortés, Lesley F. Cohen, Neil M. Alford, Johannes Lischner, Peter Petrov, Stefan A. Maier, Rupert F. Oulton

**Affiliations:** † Department of Physics, Imperial College London, London SW7 2BW, U.K.; ‡ Department of Materials, Imperial College London, London SW7 2AZ, U.K.; § Thomas Young Centre for Theory and Simulation of Materials, Imperial College London, London SW7 2AZ, U.K.; ∥ Nanoinstitut München, Chair in Hybrid Nanosystems, Faculty of Physics, Ludwig-Maximilians Universität München, Königinstrasse 10, 80539 München, Germany

**Keywords:** hot electrons, photocatalysis, plasmonics, pump−probe spectroscopy, electron lifetimes

## Abstract

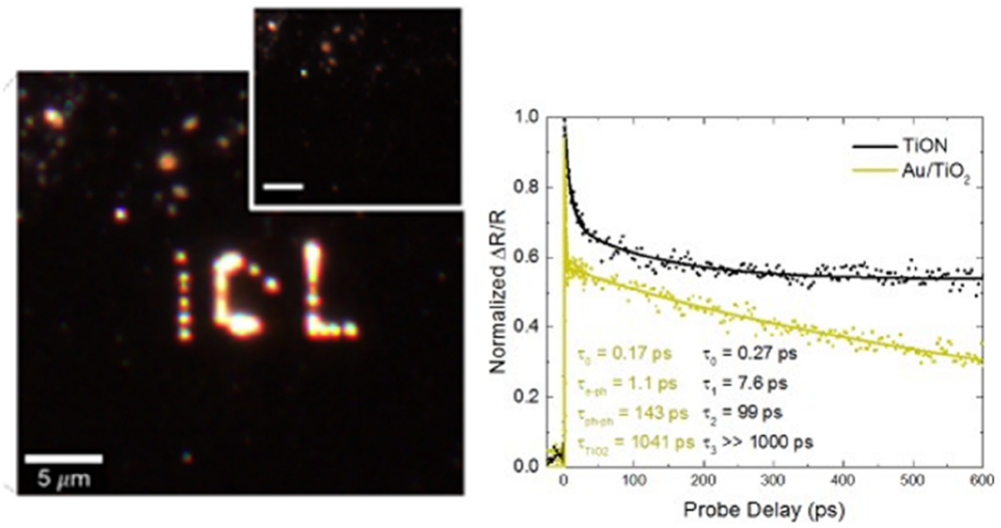

Understanding metal–semiconductor interfaces is critical to the advancement of photocatalysis and sub-bandgap solar energy harvesting where electrons in the metal can be excited by sub-bandgap photons and extracted into the semiconductor. In this work, we compare the electron extraction efficiency across Au/TiO_2_ and titanium oxynitride (TiON)/TiO_2–*x*_ interfaces, where in the latter case the spontaneously forming oxide layer (TiO_2–*x*_) creates a metal–semiconductor contact. Time-resolved pump–probe spectroscopy is used to study the electron recombination rates in both cases. Unlike the nanosecond recombination lifetimes in Au/TiO_2_, we find a bottleneck in the electron relaxation in the TiON system, which we explain using a trap-mediated recombination model. Using this model, we investigate the tunability of the relaxation dynamics with oxygen content in the parent film. The optimized film (TiO_0.5_N_0.5_) exhibits the highest carrier extraction efficiency (*N*_FC_ ≈ 2.8 × 10^19^ m^–3^), slowest trapping, and an appreciable hot electron population reaching the surface oxide (*N*_HE_ ≈ 1.6 × 10^18^ m^–3^). Our results demonstrate the productive role oxygen can play in enhancing electron harvesting and prolonging electron lifetimes, providing an optimized metal–semiconductor interface using only the native oxide of titanium oxynitride.

## Introduction

Plasmonic devices allow unprecedented control of light on the nanoscale^1^ and highly sensitive molecular detection^2^ through the increased interaction between a conductor’s free carriers and light via surface plasmon resonances. Although plasmonic modes decay on the order of tens of femtoseconds,^3^ much of the energy remains in excited carriers that relax ultimately through lattice interactions over picosecond time scales. Exploiting the energy that remains in these carriers has evolved into so-called “hot-carrier” applications. For example, the use of a Schottky barrier to collect hot electrons (holes) into the conduction (valence) band of a semiconductor has underpinned the burgeoning field of sub-bandgap photodetectors and photovoltaic devices.^4,5^ Another application involves the use of these energetic carriers in surface reduction and oxidation reactions for photocatalysis and solar water splitting.^6^ Due to the low absorption of gold in the red and infrared, nanoparticles are needed to enhance the absorption, but it comes at the cost of more expensive fabrication and the necessity of a range of particle sizes to best cover the solar spectrum. Transition metal nitrides provide a considerable advantage in such situations due to their strong broadband absorption^7^ as well as the ability to tune their electronic and optical properties by varying deposition conditions.^8^

Titanium nitride (TiN), a ceramic with tunable stoichiometry, is known to have a high free carrier density such that it exhibits optical properties similar to gold in the visible and near-infrared regimes^9−11^ but with significantly improved resilience to high temperatures.^8,12,13^ Additionally, titanium nitride has been shown to achieve enhanced hot electron harvesting relative to gold,^14−16^ and indeed it is reported that TiN has long-lived photoexcited carriers,^17^ but the physical origin of this phenomenon is poorly understood, as we show from theoretical considerations of the decay mechanisms in pure TiN. A better understanding of the material and its carrier decay dynamics holds the key to unraveling the underlying electronic processes taking place both within the material and during charge transfer to neighboring materials. Although single crystalline TiN can be epitaxially grown on specific substrates,^18^ most sputtered TiN found in practical applications contains an unavoidable amount of oxygen due to nitrogen substitution at grain boundaries.^19^ The physical properties of TiN are extremely sensitive to the substitution of oxygen within its lattice, enabling also the tuning of its optical response. Previously we have shown that titanium oxynitride (TiON) films exhibit intermediate properties between titanium nitride and titanium dioxide, including the emergence of two tunable epsilon near zero (ENZ) points.^20^ Titanium nitride and titanium oxynitride exhibit metallic behavior including high conductivity and negative real component of the permittivity. Therefore, herein we refer to these materials as metals for simplicity but note that strictly they are metal-like ceramics.

Here, we demonstrate how time-resolved pump–probe spectroscopy can be used to simultaneously investigate the electron dynamics in both metals and semiconductors as well as the specific dynamics associated with the interface between Au/TiO_2_ (metal–semiconductor) and between Au/SiO_2_ (metal–insulator). Using these as control samples, we show that titanium oxynitride (which is interfaced with its own semiconducting TiO_2–*x*_ surface oxide layer^21^) exhibits a fundamentally different recombination mechanism than that at the Au/TiO_2_ interface, showing carrier lifetimes beyond nanoseconds. By taking into account the results of density functional theory calculations and experimental material characterization (secondary ion mass spectroscopy and spectroscopic ellipsometry), we infer that the observed signal is due to an electron transfer process from the TiON into the TiO_2–*x*_, where recombination takes place with the holes residing in the TiON (as TiO_2_ is a known hole blocking layer even only several atomic layers thick^22^) through trap-assisted processes. We introduce a kinetic model to discern the underlying physical processes, which illustrates how material composition influences the trapping and recombination of the extracted carriers. Variation of oxygen content in the underlying film allows tunability of free carrier densities at the surface by an order of magnitude. With our optimized film, we can demonstrate the presence of out-of-equilibrium hot carriers at the free surface, readily available to participate in surface chemical reactions.

**Figure 1:**
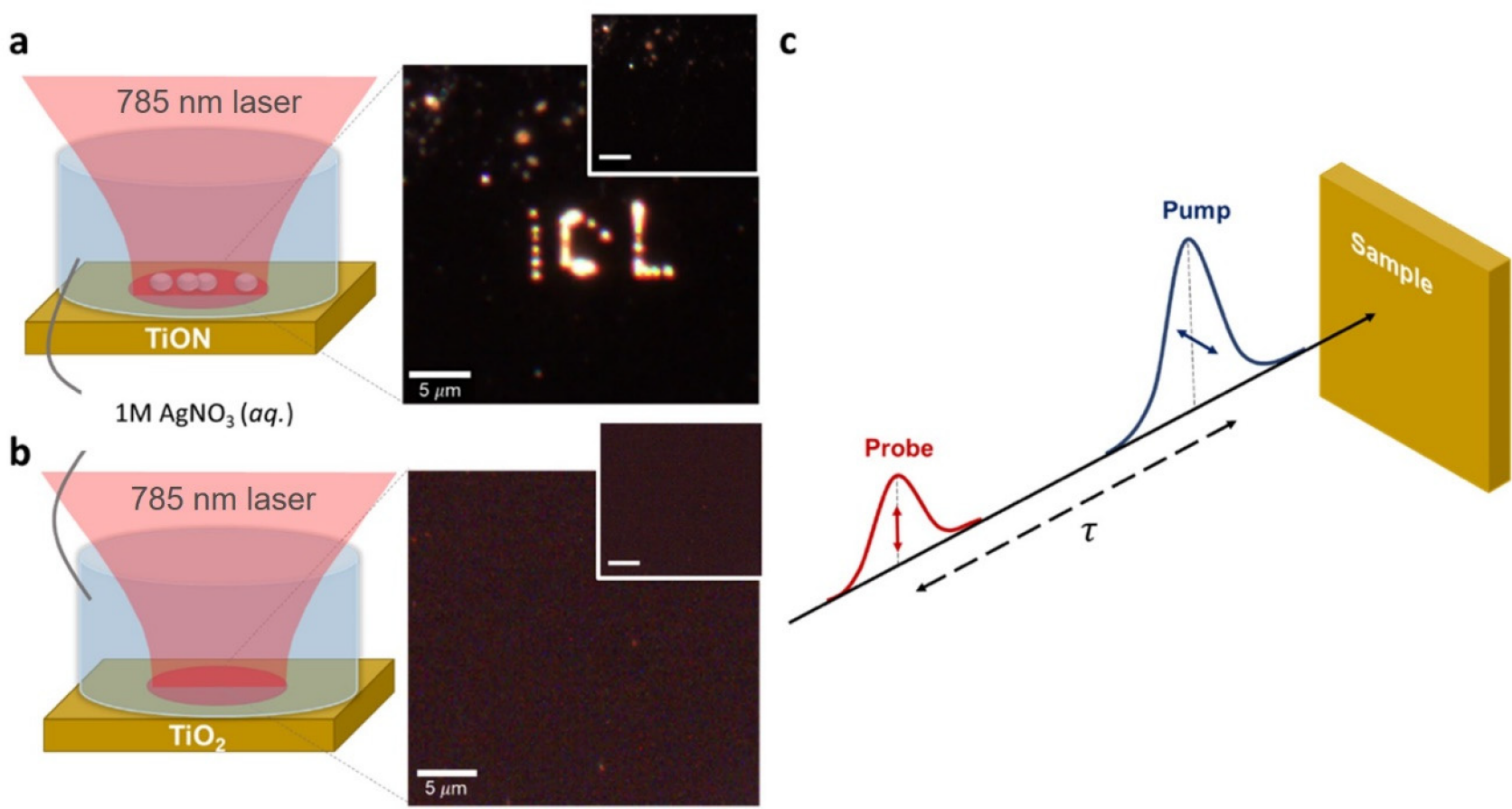
Preliminary photochemical experiments and schematics. (a) Diagram of silver reduction procedure where free carriers created via optical absorption in titanium oxynitride travel through the TiO_2_ surface oxide layer and reduce Ag^+^ ions from solution, which leads to nucleation of Ag colloids on the film surface. Dark-field images of the titanium oxynitride film surface immersed in the Ag solution prior (inset) and after laser illumination (785 nm). Selective formation of silver clusters on the surface of the film using laser excitation of electrons in the TiON can be clearly seen, forming the ICL acronym. (b) Diagram and dark-field images for the same experiment but using a TiO_2_ film as substrate. As it can be seen from the dark-field images prior to (inset) and after laser excitation, there is no silver deposition in this case. (c) Schematic diagram of pump–probe spectroscopy used throughout this work where a high power pump pulse excites carriers in a sample, the influence of which on the optical response is then measured using a time-delayed probe pulse. Scale bars correspond to 5 μm in every case.

Upon exposing TiN films to air, it is recognized that a self-limiting nonstoichiometric semiconducting titanium dioxide (TiO_2–*x*_ where *x* quantifies oxygen vacancies that may be present) surface oxide forms, protecting the film against further oxidation or damage from external contaminants.^23^ Conveniently, for sufficiently low oxygen vacancies this surface oxide is semiconducting but with a large bandgap between 3.4 and 3.6 eV for 0 < *x* < 0.3.^24^ Such metal–semiconductor interfaces are critical for many solar-based applications such as photovoltaics and solar water splitting due to the ability to separate energetic carriers that are excited with photon energies below the bandgap of the semiconductor. Separating these carriers before the electrons can thermalize with the lattice of the metal (typically on the order of several picoseconds^25^) allows for a much larger window for these carriers to be harvested or used in chemical reactions of nanoseconds or longer.^26^ The complex interlink between carrier density, mobility, and lifetime can be affected by oxygen vacancies, defects, etc. This could significantly impact the efficiency and rate of chemical reactions.^27,28^ In the case of TiN and TiON, the role of the surface oxide is not clear as it physically separates the absorbing layer and the molecules participating in the reaction. As a first step, we investigate the hot electron reduction of silver ions^29−31^ from a 1 mol/L solution of AgNO_3_ in degassed DI water by a TiON film (Figure 1a) and a sputtered TiO_2_ control (Figure 1b). Silver reduction is a well-known reaction on metal oxide semiconductors in the presence of water,^32,33^ and it also has the advantage that Ag deposition can be clearly visualized by dark-field microscopy (as shown in Figure 1a). The TiO_2_ sample was illuminated at 7 mW with a 785 nm CW laser for over 1 min with no formation of Ag on the surface. However, in TiON laser exposure for 20 s at 6 mW allowed selective writing by reduction of silver ions on the surface of the film as seen in Figure 1a. This suggests that even in the presence of the surface oxide layer, electrons with sufficient energy to participate in this reaction reach the surface. Both films behave differently under the same reaction conditions, and as such, this requires a closer investigation of the ultrafast electron dynamics in the film.

## Results and Discussion

**Figure 2:**
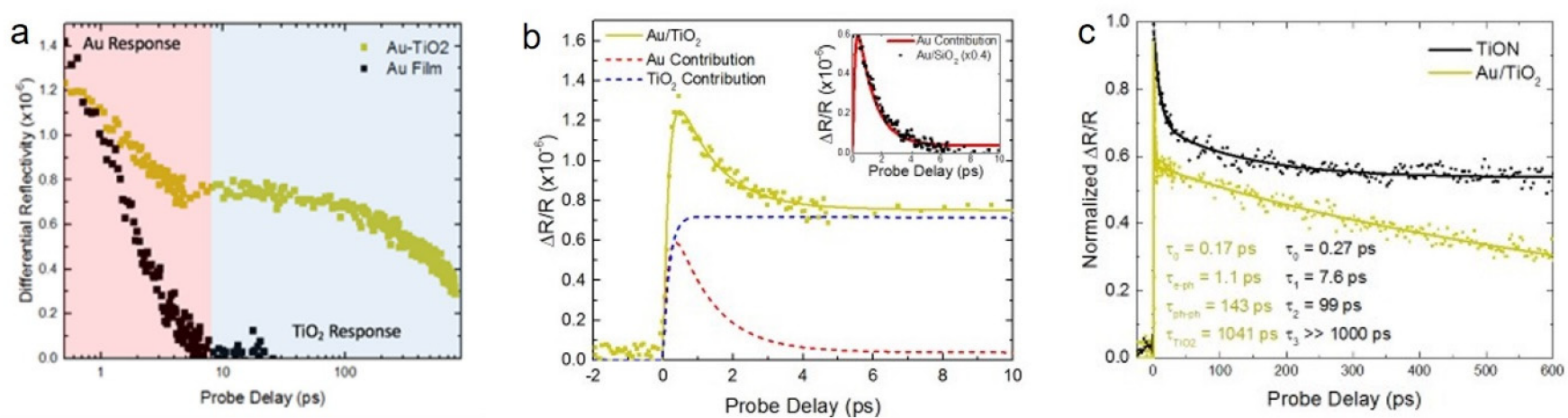
Time-resolved pump–probe spectroscopy to investigate dynamics at a metal–semiconductor interface. (a) Semilogarithmic plot of the time-resolved differential reflectivity of Au/TiO_2_ (metal–semiconductor) and Au/SiO_2_ (metal–insulator). The Au response (red shaded region) is clearly delineated from the TiO_2_ response (blue shaded region) confirmed by the Au/SiO_2_ control sample, which only has the Au response. (b) Using a combination of the two-temperature model and metal–semiconductor recombination, the two contributions are separated showing long-lived electrons remaining in the TiO_2_ conduction band. The inset shows the fitted Au contribution alongside the Au/SiO_2_ sample (scaled by a factor of 0.4) showing very good agreement. (c) Differential reflectivity signals of TiON and Au along with the fitted lifetimes. Beyond 300 ps, the TiON signal is constant, in contrast to the typical Schottky barrier recombination lifetime seen in the Au/TiO_2_ suggesting a different recombination mechanism.

**Figure 3:**
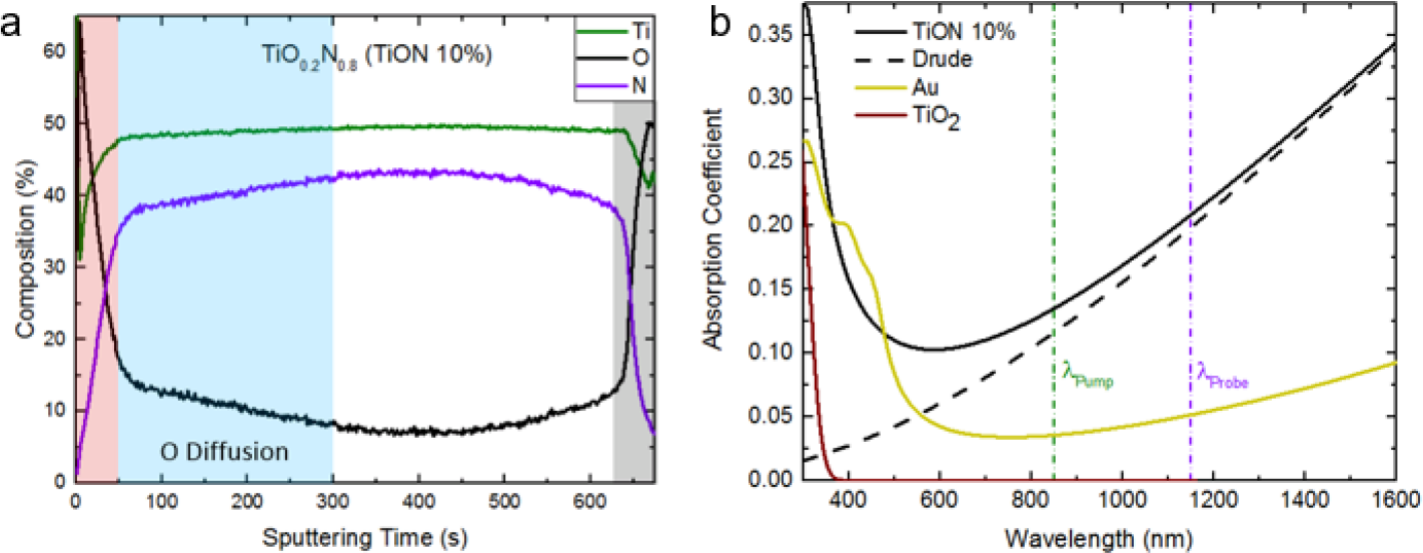
Titanium oxynitride material characterization. (a) Time-of-flight secondary ion mass spectrometry (ToF-SIMS) characterization of the elemental composition profile of the film determined to be TiO_0.2_N_0.8_, termed TiON 10%. The TiO_2–*x*_ surface oxide (red shaded region) and SiO_2_ substrate (gray shaded region) form the two boundaries of the TiON 10% film. Oxygen diffusion into the film is clearly visible below the surface oxide (blue shaded region). (b) Absorption spectra of TiON and Au measured by spectroscopic ellipsometry and fitted to a Drude–Lorentz model along with the pump (green) and probe (purple) wavelengths used in this study. The free electron (Drude) contribution of TiON is shown with the dashed line, and TiO_2_ is shown as a reference to show that there is no surface oxide absorption in the TiON film.

To investigate the dynamics of the optically excited electrons, we measure the time-resolved differential reflectivity using pump–probe spectroscopy with two <200 fs pulses (shown schematically in Figure 1c). A 5 mW, 850 nm pump pulse excites carriers in the material, and we then monitor the change in refractive index (Δ*n*) by measuring the reflectivity of a time-delayed 150 μW, 1150 nm probe pulse. These conditions were maintained for all pump–probe measurements. The pump and probe wavelengths were selected partly for convenience in operating the laser system. The pump wavelength was required to be below the bandgap of TiO_2_ to ensure hot carriers would be generated in the TiN only (see Figure 3b). We note that stronger differential reflectivities were observed for small pump–probe wavelength separations. We begin by characterizing two continuous film control samples: 50 nm of gold on fused quartz (Au/SiO_2_) and 50 nm of gold deposited on amorphous TiO_2_ (Au/TiO_2_), the latter of which is known to exhibit a slow decay due to carrier separation at the Schottky barrier.^26^Figure 2a shows the time-resolved differential reflectivity of both samples on a semilogarithmic plot. The Au/SiO_2_ sample shows a rapid decay within 10 ps, which can be fitted to the two-temperature model,^25^ describing the interaction between high-energy electrons and low-energy phonons via exchange of thermal energy. This decay can be approximated as a biexponential function with decay lifetimes associated with electron–phonon and phonon–phonon scattering, herein represented as τ_e__–ph_ and τ_ph__–ph_, respectively. However, in addition to the Au response, the Au/TiO_2_ sample exhibits a long-lived decay associated with free electrons in the conduction band of the TiO_2_. The semiconductor contribution to the differential reflectivity is a direct measure of the electron harvesting efficiency as the signal is proportional to the free carrier concentration *N*_FC_.^34^ At normal incidence
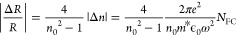
where *e* is the charge of an electron, *n*_0_ is the unperturbed refractive index of TiO_2_, m* is the effective mass, and ω is the frequency of the probe beam.

**Figure 4:**
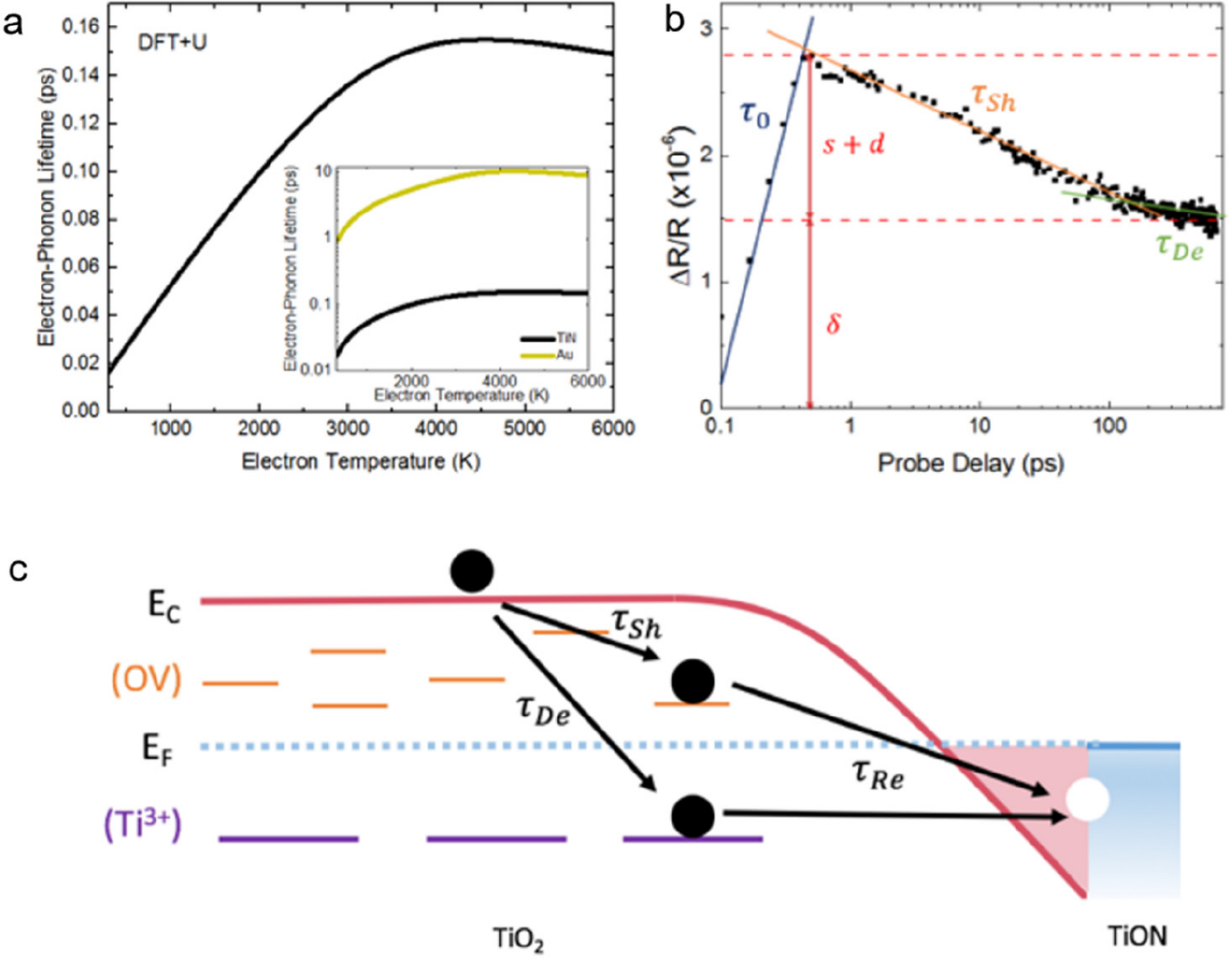
Theoretical investigation of the TiON–TiO_2**–***x*_ interface and the trap-assisted recombination model. (a) Ab initio DFT+U calculations of the electron–phonon scattering time of TiN. The strong electron–phonon coupling of TiN results in thermalization with the lattice within the resolution of our system. Inset shows a comparison of calculated Au and TiON lifetimes with Au showing 2 orders of magnitude longer lifetimes. (b) Proposed trap-assisted recombination model at the TiON–TiO_2–*x*_ interface with the associated lifetimes observable in our measurements associated with shallow trap occupation (τ_Sh_), deep trap occupation (τ_De_), and recombination (τ_Re_). (c) Experimental data showing the shallow and deep trap occupation lifetimes along with the exponential rise time of the signal (τ_0_) and the amount of energy lost to occupying traps (*s* + *d*) and the amount remaining in the free carriers (δ).

By decomposing the individual contributions of the metal and semiconductor, we have a means of evaluating the electron extraction efficiency by purely optical means. Figure 2b shows the differential reflectivity of the Au/TiO_2_ sample fitted to a sum of three exponential decay functions over the first 10 ps. Here, the hot carrier relaxation in gold follows a single exponential, while the recombination of carriers transferred to the TiO_2_ is fit with a biexponential decay, following the model described later and illustrated in Figure 4. Using the fitted data, the signal is decomposed into separate Au and TiO_2_ signals showing that beyond 5 ps the signal is dominated by the TiO_2_ response. Using the maximum of the TiO_2_ contribution, we can estimate the extracted carrier concentration to be 2.8 × 10^18^ m^–3^. In the inset of Figure 2b we show the fitted Au contribution along with the Au/SiO_2_ measurement scaled by a factor of 0.4. The similarity of the two decays reinforces the validity of our decomposition. With our control samples well understood, we now look to compare the Au/TiO_2_ behavior with that of TiON/TiO_2_. Because it is only the free electrons in TiO_2–*x*_ injected from TiON that absorb the 1150 nm probe pulse,^35^ we know that we only measure the free electrons, which are capable of participating in chemical reactions. The two normal differential reflectivity signals are plotted alongside the parameters extracted from the fits in Figure 2c. There is a striking difference observed between the two samples, particularly beyond 200 ps where the recombination in the TiON sample appears to bottleneck. This difference at longer time scales suggests that the recombination process merits much more detailed material analysis.

First we examine how the elemental composition varies along the depth of the films using time-of-flight secondary ion mass spectrometry (ToF-SIMS). Using the composition of the material at the midpoint between the surface (*t*_sputtering_ = 0 s) and substrate (*t*_sputtering_ = 625 s), we determine the film shown in Figure 2 to be nearly stoichiometric TiO_0.2_N_0.8_ (Ti: 50%; N: 40%; O:10%, which we name TiON 10% to reflect the relative oxygen content of the film), shown in Figure 3a. The film displays a sharp increase of oxygen and decrease of nitrogen at the surface, confirming the presence of interfacial TiO_2–*x*_. This likely forms an Ohmic contact between the conductive TiON film and semiconducting TiO_2–*x*_ (individual and interfacial band diagrams are shown in Supporting Information Section S7). Below that there is a rapid onset of nitrogen content within the first 50 s of sputtering followed by a secondary slower increase of nitrogen until it stabilized around 250 s. The latter transitional layer is a result of oxygen diffusing into the film and shows a decrease with depth due to the energy barrier for oxygen at the surface to penetrate into the bulk of the film.^36,37^ It is thought that the randomness associated with the oxygen substitution has the potential to increase disorder at the interface and could result in a higher density of oxygen vacancies.^38^ To investigate the absorptive properties of the film, we use spectroscopic ellipsometry fitting to a Drude–Lorentz model with an oxide layer to determine the permittivity (Supporting Information Section S1). Using this, we calculate the absorption coefficient plotted in Figure 3b along with that of Au and the fitted Drude (free carrier) contribution to the TiON. As TiO_2–*x*_ only absorbs photons above its bandgap energy (wavelengths below 400 nm) shown by the dark red curve in Figure 3b, we conclude that the absorption at 850 nm is via the free carrier absorption in the underlying TiON 10% film.

As we are looking at a novel material system, little is known about the interactions between electrons and phonons within the material. The previous demonstration of a long-lived differential reflectivity signal in TiN films was interpreted in terms of weak electron–phonon coupling^17^ The temperature-dependent electron and phonon coupling of TiN can be modeled using ab initio DFT+U calculations,^39^ which we present in Supporting Information Section S3. Remarkably, the results reveal that the pure TiN electron–phonon coupling constant is 2 orders of magnitude greater than that of our calculated values of Au, consistent with known values of Au.^40^ The corresponding electron–phonon lifetimes are shown in Figure 4a, which are on the order of 100 fs or faster, consistent with other theoretical calculations and experimental observations.^41,42^ To model the disordered TiON system, the virtual crystal approximation could be considered to calculate the electron relaxation time.^39^ These calculations predict that the relaxation time is almost independent of the oxygen content at low densities. The inset of Figure 4a compares the calculated electron–phonon lifetimes of TiN and Au, and although this agrees very well with the calculated τ_e__–p_ of our control sample, the calculated lifetimes of TiN cannot explain the long experimentally observed lifetimes. Care must be taken to distinguish this from the electron–electron scattering time (τ_e__–e_), which is known to be on the order of femtoseconds for gold.^43^ However, in gold films, the electron–phonon scattering time (τ_e__–p_) has been observed to be on the order of picoseconds.^25^ Furthermore, as time scales this fast are below the temporal resolution power of our setup, the implication is that the entire observed signal must originate from the refractive index change associated with the occupation of the conduction band states in the TiO_2–*x*_ surface oxide layer.

Titanium nitride, and by extension titanium oxynitride, is a very unique material with respect to electron harvesting. There exist few previous reports where efficient electron harvesting occurs across an Ohmic contact.^10,14−16^ In the case of Schottky contacts, charge separation is maintained by the intrinsic electric field; however, it is not clear how charge separation is achieved in our system. With the existing studies, our experimental observations, and DFT results in this work, we put forth the following explanation. In a typical metal the electron thermalization is explained with the two temperature model^44^ where a nonthermal population thermalizes with other electrons via electron–electron scattering until an elevated temperature Fermi distribution is reached. The electrons then cool by heating the lattice via electron–phonon scattering, eventually reaching a bottleneck when the two temperatures equate where the electrons can no longer transfer excess energy to the phonons. Subsequent cooling then follows the rate of heat dissipation through the material and to the environment. With the ultrafast electron–phonon thermalization in TiN/TiON, this bottleneck is reached on the same time scale as the electron–electron scattering, effectively trapping electrons in the high-energy states. The injection of electrons into TiO_2–*x*_ is then simply diffusion into the lower energy unoccupied conduction band states. As the thermal conductivity of TiN is known to be very low compared to most metals,^45^ the cooling of the electrons in the TiN is extremely slow, which gives the harvested electron sufficient time to be trapped within the TiO_2–*x*_ as observed experimentally. Further photochemical and photocurrent measurements can be performed to confirm the exact mechanism.

Because both electron injection into TiO_2–*x*_ and electron relaxation in TiON occur within the pulse width of the pump beam, what is required here is a realistic description of the subsequent relaxation and recombination channels of free carriers in TiO_2–*x*_. We propose that the recombination occurs via trap states in the TiO_2–*x*_ and at the interface with TiON and that the recombination is slowed because of the saturation of said states and slow detrapping times. In TiO_2_ there are two sources of trap states: oxygen vacancies (OV) forming traps close to the conduction band edge (shallow traps) and Ti^3+^ forming deep traps within the bandgap. Similar to what is seen in the dye-sensitized titanium dioxide,^46,47^ we observe the occupation of the trap states occurring faster than recombination back into the metal until a quasi-equilibrium is reached when the trap states are fully occupied. Following this, the subsequent decay is known to be on the order of nanoseconds to milliseconds as it follows the rate of detrapping to recombine with the hole remaining in the metal. Figure 4b shows a schematic of the proposed recombination model and the associated lifetimes as is typically associated with shallow trapping (τ_Sh_) and deep trapping (τ_De_).^46,48^Figure 4c shows a semilogarithmic plot of the TiON 10% data with the proposed lifetimes clearly delineated. In addition we show τ_0_, which is a simple fit to the exponential rise of the signal associated with both the overlap of pump and probe pulses convolved with the rise associated with the occupation of the conduction band states.

The density of free electrons in the conduction band of TiO_2–*x*_ is described by *N*_FC_(*t*) and decays as the free electrons occupy the shallow (*N*_Sh_(*t*)) and deep (*N*_De_(*t*)) traps. Thus, the temporal evolution of the free carriers is described by *N*_FC_(*t*) = *N*_FC_(0) – *N*_Sh_(*t*) – *N*_De_(*t*). The rate of occupation of these trap states is proportional to the availability of trap states, decreasing with increasing occupation. To quantify this, we describe maximum occupation values, *S* and *D*, for shallow and deep traps, respectively. We then denote the rates of proportionality for the shallow and deep states as *k*_Sh_ and *k*_De_ leading to the following two differential equations:
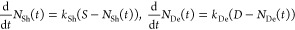
This has a simple solution, resulting in the biexponential decay:
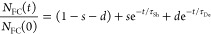
where *s* = *S*/*n*_Free_(0) and *d* = *D*/*n*_Free_(0). We denote the constant value 1 – *s* – *d* as δ, which characterizes the residual electron occupation after the trap states are fully occupied. With the relationship between the free carrier concentration and differential reflectivity already established above, we can now relate the decays observed in TiON to the underlying physical processes. As such an approach considers the electron distribution to be in quasi-equilibrium, it is not valid on time scales below a few tens of femtoseconds.

**Figure 5:**
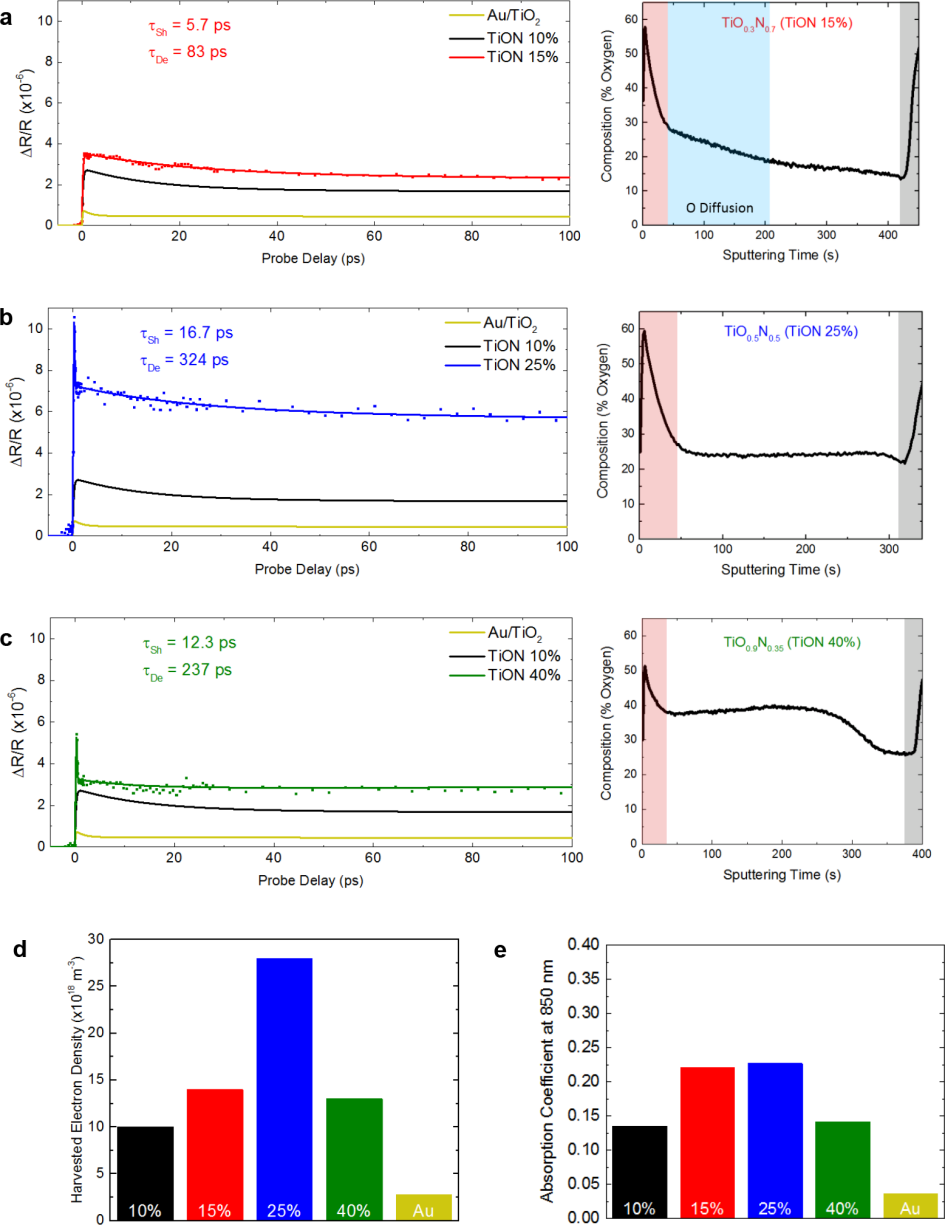
Optimization of hot electron injection by tailoring the oxygen content in TiON thin films. (a–c) Differential reflectivity following a 5 mW pump pulse (left) and oxygen composition (right) measured with ToF-SIMS for films with increasing oxygen content TiON 15% (a), TiON 25% (b), and TiON 40% (c). Each film exhibits a higher differential reflectivity than both the Au/TiO_2_ and TiON 10% films over the entire temporal range. With sufficiently high oxygen included in the TiON films (25% and 40%) no postdeposition oxygen diffusion is detected as is seen in the TiON 10% and TiON 15% films. This more uniform interface results in more energetic electrons reaching the TiO_2–*x*_ interface and the emergence of an ultrafast peak. (d) Maximum free carrier concentration in the TiO_2–*x*_ determined via the fitting of differential reflectivity measurements. (e) Absorption coefficient at the pump wavelength (850 nm) for each film. It is clear that the strong enhancement in electron harvesting observed in TiON 25% cannot be explained simply by an increase in absorption.

In TiON 10% we observe the shallow trapping to be relatively quick compared to pure TiO_2_, τ_Sh_ = 7.6 ± 0.8 ps, compared to 29.8 ps in pure TiO_2_,^48^ suggesting a significant amount of oxygen vacancies in this film. Furthermore, the fitted deep trap lifetime τ_De_ = 99 ± 12 ps is lower than pure TiO_2_ (471 ps^48^) but is approximately the same as metal-doped TiO_2_.^48^ Thus, this is consistent with the increase in deep traps (Ti^3+^) that have not reacted with oxygen. In order to explore the model further, we study three additional TiON films with a systematically increasing oxygen content: TiON 15%, TiON 25%, and TiON 40%. We anticipate that the increased oxygen content in the underlying TiON films will react with the Ti^3+^ ions and decrease both shallow and deep traps, resulting in an increase to the observed lifetimes. Figures 5a–c show the oxygen content profile of each of the three additional films measured by ToF-SIMS and the associated differential reflectivity signals compared to the TiON 10% and Au/TiO_2_ samples. We note the difference in sputtering time of the TiN films is a result of the decreasing hardness and decreases monotonically with increasing oxygen (Supporting Information Section S6). By increasing the oxygen content slightly to 15% (Figure 5a), we observe a slightly larger differential reflectivity, but similar trapping lifetimes (τ_Sh_ = 5.7 ± 0.8 ps, τ_De_ = 83 ± 12 ps). This is to be expected as we still observe oxygen diffusion into the film, suggesting a porous surface oxide (shaded region). The effect of oxygen on the metallic behavior is characterized through the dielectric permittivity shown in Supporting Information Section S1 measured by spectroscopic ellipsometry.

When increasing the oxygen further to 25%, we observe a distinct change in behavior of both the oxygen profile and differential reflectivity as seen in Figure 5b. We observe uniform oxidation throughout the TiON film, suggesting the TiO_2–*x*_ formed is uniform and effectively blocks the further diffusion of oxygen into the film. This is also reflected in a substantial increase in the differential reflectivity signal, associated with more efficient electron extraction and the extended lifetimes of the TiON 25% (τ_Sh_ = 16.7 ± 3.4 ps, τ_De_ = 324 ± 70 ps), which now approach the values for pure TiO_2_. This is a direct demonstration of using the oxygen content in TiON to tune the electron relaxation dynamics in the adjacent semiconductor to make it more favorable for hot electron applications. In this case we also observe a distinct ultrafast decay not present in either the TiON 10% and TiON 15%, which will be analyzed in detail in the subsequent section. The heavily oxidized TiON 40% exhibits similarly long lifetimes (τ_Sh_ = 12.3 ± 4.6 ps, τ_De_ = 237 ± 130 ps) as well as the ultrafast peak. However, the magnitude of the differential reflectivity signal is weaker than that of TiON 25% due to the less metallic behavior. In Figure 5d we show the estimated harvested carrier concentration using the fitted differential reflectivity data measured for each of the films. We estimate the extracted carrier concentrations to be *N*_FC_^10%^ = 1.0 × 10^19^ m^–3^, *N*_FC_^15%^ = 1.4 × 10^19^ m^–3^, *N*_FC_^25%^ = 2.8 × 10^19^ m^–3^, and *N*_FC_^40%^ = 1.3 × 10^19^ m^–3^ for the four TiON films. The superiority of TiON 25% is clear in that it is the most efficient interface to harvest electrons, and the harvested electrons remain in the semiconductor conduction band for longer compared to the other films. Although shallow trap absorption in TiO_2_ is known to be much weaker than the free carrier absorption at these wavelengths, some absorption occurs.^49^ This may result in a slight overestimation of the harvested carrier density, but as this process is inefficient and also dependent on the shallow trap density, it is assumed to be insignificant. The response of TiO_2–*x*_ was measured directly in Supporting Information Section S5 and shown to be negligible.

**Figure 6:**
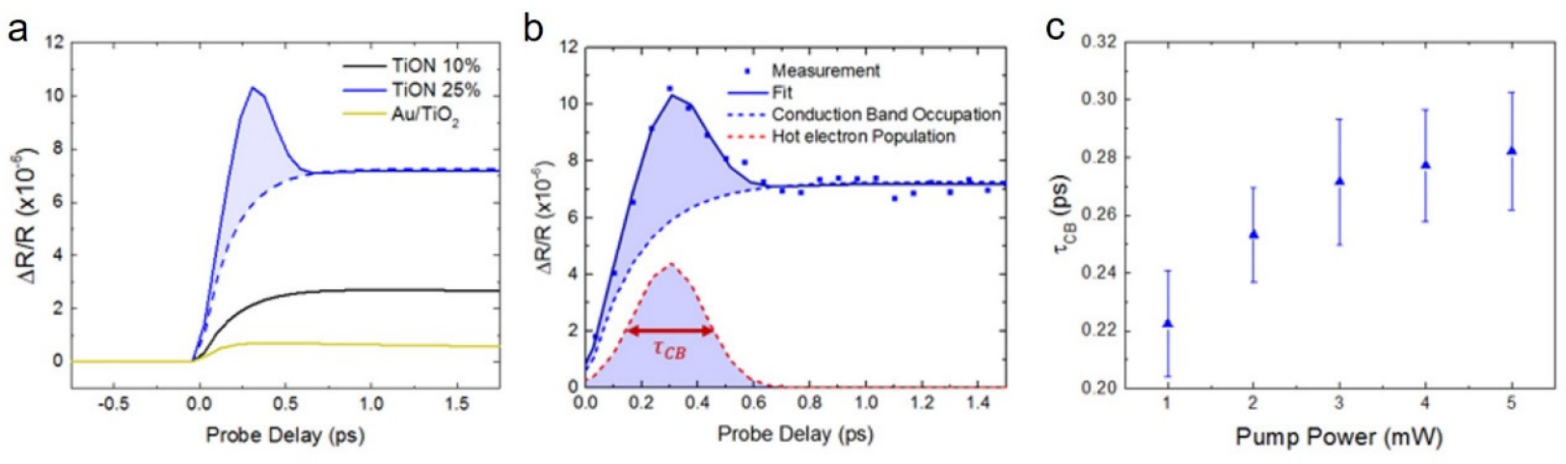
Hot electron dynamics in the surface oxide of TiON 25%. (a) Differential reflectivity measurements of Au/TiO_2_, TiON 10%, and TiON 25% over the first 1.75 ps. TiON 25% exhibits an additional ultrafast peak (shaded region) attributed to hot electrons in the TiO_2–*x*_ relaxing to the conduction band minimum. (b) Decomposition of the TiON 25% signal into conduction band electron occupation (blue dashed line) and hot electron population (red-dashed line). The lifetime of the hot electron distribution (τ_CB_) describes the electron–phonon scattering time in the TiO_2–*x*_. It is described by the fitted full width at half-maximum (FWHM) of the signal showed by the red arrow. (c) Power dependence of τ_CB_, showing a slower relaxation with increasing power (and thus increased electron temperature) characteristic of a hot electron population. Error bars denote standard deviation of the fitted data.

Figure 5e shows the absorption coefficient for each of the materials at 850 nm. The advantages of the TiON 25% film cannot be explained simply by an increase in absorption, which is comparable to TiON 15%. Furthermore, the significant increase in absorption of TiON 15% over TiON 10% only results in a modest increase in differential reflectivity. Thus, the injection behavior between these films must differ in a way that favors transfer into the semiconductor. We posit that the low-defect interface of TiON 25% and 40% facilitates the direct injection of hot electrons into the conduction band of the TiO_2–*x*_, which subsequently relax to the conduction band minimum via electron–phonon scattering. To investigate this injection behavior more closely, we have fit the ultrafast peak as a Gaussian-shaped contribution shown as the shaded regions of TiON 25% in Figure 6a,b. Using the height of the fitted Gaussian, we estimate the hot carrier concentration in the oxide layer to be 1.6 × 10^19^ m^–3^. We estimate the lifetime, τ_CB_, of this population as the full width at half-maximum (FWHM) of the fitted Gaussian shown schematically in Figure 6b. This lifetime serves as an estimate of the electron–phonon scattering time within the TiO_2–*x*_. Furthermore, the pump power dependence of τ_CB_ shown in Figure 6c indicates dynamics that depend on the electron temperature, as a higher pump power should suggest a higher electron temperature. It is known that hot electron effects show distinctive temperature-dependent relaxation dynamics^50^ due to the relationship with volumetric heat capacity and electron–phonon coupling (Section S3).^50^ This power-dependent behavior is not observed in the subsequent two lifetimes (τ_Sh_ and τ_De_) as the electrons have lost their excess energy and remain at the conduction band minimum (Section S4). It should be emphasized that this is not possible in the more metallic TiON 10% and TiON 15%, as the high disorder of the interface scatters the energetic electrons limiting transfer to the conduction band.^51^

## Conclusions

In this work, we have closely examined the critical role that oxidation plays in hot carrier separation of titanium oxynitride thin films. We introduce an interfacial charge transfer model that provides a framework for describing the interplay between the strongly absorptive metallic layer and the omnipresent surface oxide layer. Hot electron injection into the conduction band of the surface titanium dioxide layer is achieved by engineering the interfacial oxygen defect states. Reducing the density of the traps in the surface oxide and at the interface also slows the energy loss over the first few hundred picoseconds. The subsequent recombination with the metal occurs on the time scale of nanoseconds or longer, confirming that we have trap-mediated carrier separation. The partially oxidized titanium oxynitride film (TiON 25%) shows the most promise for use in hot carrier applications as it exhibits much more efficient carrier separation (as well as an estimated hot carrier concentration of 1.6 × 10^19^ m^–3^ at the interface) and retains the greatest portion of its initial energy over the time period measured. Our work affirms the indispensable role that titanium oxynitride can play in the future development of plasmonic and hot carrier applications. Such a hybrid material system has improved versatility, using only its natural oxidation tendencies to achieve efficient extraction of electrons over a wider range of energies than is possible with comparable Schottky barrier systems.

## Experimental Section

### Deposition

Titanium nitride films are deposited on fused quartz substrates using reactive RF magnetron sputtering from a titanium target in a N_2_/Ar (30% N_2_) plasma. 50 nm of each film (TiON 10%, TiON 15%, and TiON 25%) was deposited at high temperature (600 °C). For the TiON 10%, oxygen contamination during growth was minimized (<5 × 10^–9^ mbar O_2_ partial pressure) by running a 1 h Ti presputter (Ti is known to be a good O_2_ getter). A shorter presputter for the 50 nm TiON 15% was performed in order to get an intermediary O_2_ residual level in the sputtering chamber, and the film was grown at high temperature (600 °C). No presputtering was done for the deposition of the TiON 25% film. 100 nm of the TiON 40% film was deposited at room temperature with no presputter onto a fused quartz substrate. More information on the growth methods can be found in ref (18).

### Silver Reduction Experiments

To stimulate the study of the dynamics of hot electron transfer from Ti(O)N into the conduction band and trap states of the TiO_2_ layer, we tested the photoinduced reduction of Ag^+^ from a 1 M AgNO_3_ aqueous solution on the TiO_2_ surface. We placed the sample in a transmission dark-field microscope with illumination from below and the solution drop-casted on top. Ag deposition was accomplished under 785 nm laser excitation from the top through a 63× water immersion objective (NA = 1.0). The Ag deposition process could be follow in real time by dark-field imaging. For laser powers of 6 mW we observed growth of Ag particles at the laser focus spot after excitation durations up to 30 s. The absence of bubble formation during excitation infers a local temperature below 400 K which should not allow for driving the reaction thermally. As a control experiment we tested Ag reduction on a 200 nm thick TiO_2_ film sputtered on a glass substrate under the same conditions. Here we could not observe Ag particle growth also for a laser power of 7 mW and excitation durations of 60 s. From this comparison we conclude that the transferred and trapped electrons can diffuse to the surface and can be used for surface photochemistry under excitation energies way below the bandgap of TiO_2_.

### Density Functional Theory Calculations

We performed density functional theory (DFT) calculations of the electronic states, phonons, and electron–phonon matrix elements using the Quantum Espresso software package.^52^ We used ultrasoft pseudo potentials (USPP) and the BLYP exchange-correlation functional. Additional details can be found in Supporting Information Section S3.

### Characterization

#### Time-of-Flight Secondary Ion Mass Spectrometry

Using an ION-TOF TOF.SIMS 5 instrument, a focused ion beam of Bi_1_^+^ ions is used to ablate the sample at a fixed power. The charged ions ejected from the surface are then collected and analyzed based on their mass-to-charge ratio to determine the constituent molecules.

#### Spectroscopic Ellipsometry

Using a variable-angle JA Woollam VASE ellipsometer, the optical properties of the films used in experiments were determined using a Drude–Lorentz model. Fitting was performed using a Levenberg–Marquardt algorithm to minimize the mean-squared error (MSE). The fitted parameters of each film are presented in Supporting Information Section S1.

### Differential Reflection Measurements

Using a Chameleon Ultra II Ti:sapphire laser, an 850 nm pump pulse with temporal width below 200 fs is generated and a proportion used to produce a lower energy 1150 nm probe pulse using an optical parametric oscillator (OPO). The power of the pump pulse varied between 1 and 5 mW, but the probe is fixed at 125 μW. A motorized stage on the probe line allows for the delay between the two pulses to be controlled in steps as small as 30 fs. Using a mechanical chopper to modulate the pump beam and a photodetector and lock-in amplifier on the probe beam, time-resolved differential reflection measurements are extracted directly. A biexponential decay function is used to fit the experimental data. Fitting is done using the Levenberg–Marquardt algorithm, and the quality of the fit is determined using the adjusted *R*^2^ value, explained in Supporting Information Section S2.

## Data Availability

Any code used in the measurement and analysis of the acquired data is available from the corresponding author upon request.

## Supporting Information Available

Titanium nitride film ellipsometry, evaluation of fit accuracy for TiN ellipsometry, ab initio calculations of hot carrier thermalization in TiN, pump-power invariance of trapping lifetimes, substrate dependence of electron harvesting, sputtering time vs oxygen content for SIMS samples, schematic band diagrams of TiO_2_ and TiN (PDF)
