# Supplementary material for: Optimizing Hot Electron Harvesting at Planar Metal–Semiconductor Interfaces with Titanium Oxynitride Thin Films
Source: ACS Appl Mater Interfaces. 2023 Jun 12;15(25):30417–26. doi: 10.1021/acsami.3c02812 (PMC10316319; doi:10.1021/acsami.3c02812)
Supplement: Supplementary file 1 — am3c02812_si_001.pdf [file am3c02812_si_001.pdf]

SUPPORTING INFORMATION

Optimizing Hot Electron Harvesting at Planar Metal-semiconductor Interfaces with Titanium Oxynitride Thin Films

Brock Doiron[A], Yi Li[A,D], Andrei Mihai[B], Stefano Dal Forno[A], Sarah Fearn[B], Ludwig Hüttenhofer[D], Emiliano Cortés[D], Lesley F. Cohen[A], Neil M. Alford[B], Johannes Lischner[B,C], Peter Petrov[B], Stefan A. Maier[D,A], Rupert F. Oulton[A]*

[A] Department of Physics, Imperial College London, London, SW7 2BW, UK
[B] Department of Materials, Imperial College London, London, SW7 2AZ, UK
[C] Thomas Young Centre for Theory and Simulation of Materials, Imperial College London, London, SW7 2AZ, UK
[D] Nanoinstitut München, Chair in Hybrid Nanosystems, Faculty of Physics, Ludwig-Maximilians Universität München, Königinstrasse 10, 80539 München, Germany*

Corresponding Author Email: r.oulton@imperial.ac.uk

S1 Titanium Nitride Film Ellipsometry

Spectroscopic ellipsometry data is fitted to a Drude-Lorentz oscillator with a TiO_2 surface layer. The model parameters and oxide thickness are fitted using the Levenberg-Marquardt Algorithm as described in Supplementary Section S3. The epsilon-near-zero (ENZ) wavelength indicates the crossover wavelength from dielectric to metallic behavior and is the point where the real part of the permittivity crosses from positive to negative. This gives an intuition into the operation range of surface plasmon polariton (SPP) and localized surface plasmon resonance (LSPR) applications. For the most oxidized film (TiON 40%), it remains positive over the entire wavelength range in question and so no ENZ point is determined. Next, the energies of the two interband transitions are shown, agreeing well with previous estimates for titanium nitride[1], except for the TiON 40% film. This suggests that the inclusion of oxygen in TiON 10%, 15% and 25% does not have a notable effect on the band structure. The high-frequency permittivity (ϵ_∞) is also given, accounting for high-frequency modes not included explicitly as Lorentz oscillators. The unscreened plasma frequency (without the Coulomb screening effects of the valence electrons) is given in terms of electron-volts (eV). This is the best indicator of the metallic behavior of the material as is proportional to the square root of the conduction electron density (N) through the following expression:

$$E_P^{US} = \hbar \sqrt{\frac{Ne^2}{m^* \epsilon_0}}$$

where e is the charge of the electron, m^* the electron effective mass and ϵ_0 the vacuum permittivity. Finally, the Drude loss term, which characterizes the time between electron collisions, is given in femtoseconds accounting for electron scattering from impurities, phonons and other electrons.

	**TiO_2 Thickness (nm)**	**ENZ (nm)**	**Interband 1 (eV)**	**Interband 2 (eV)**	ϵ_∞	**E_P^{US} (eV)**	**Drude Loss Term (fs)**	**MSE**
TiON 10%	6.4	495	5.61	3.96	1.82	5.7	9.4	3.3
TiON 15%	6.8	565	5.60	4.07	2.14	5.3	4.0	6.8
TiON 25%	10.2	625	5.62	3.75	2.36	5.0	2.7	2.9
TiON 40%	4.9	N/A	4.34	1.17	3.02	N/A	2.6	9.8

Table S1 | Drude-Lorentz fitting parameters. *The fitted parameters of the spectroscopic ellipsometry measurements to a Drude Lorentz model with two Lorentz oscillators fitted using a Levenberg-Marquardt algorithm to minimize the mean squared error (MSE).*

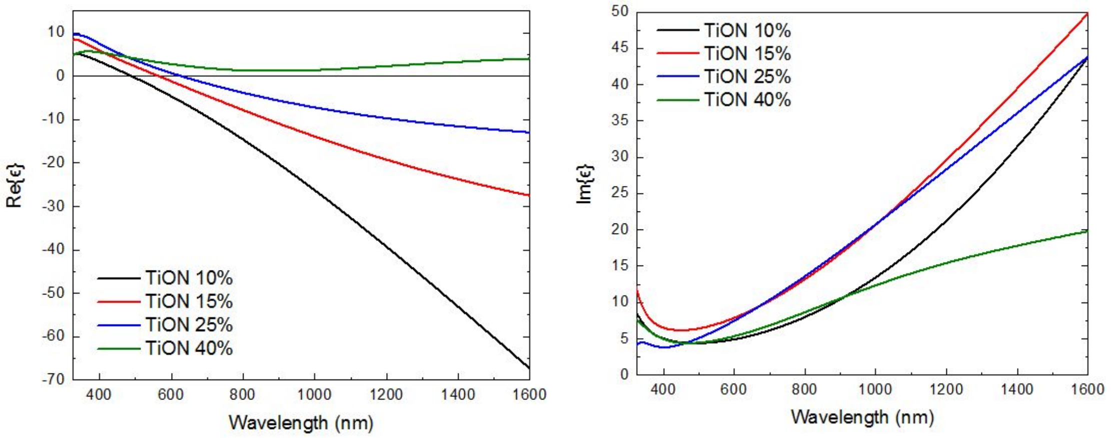

Figure S1 | Optical properties of TiON thin films. *The real (left) and imaginary (right) parts of the dielectric permittivity described by the Drude-Lorentz fitting parameters above.*

[1] Patsalas, P., Kalfagiannis, N. & Kassavetis, S. Optical properties and plasmonic performance of titanium nitride. *Materials (Basel).* **8,** 3128–3154 (2015).

S2 Evaluation of the Fit Accuracy

The Levenberg-Marquardt Algorithm is used to fit to fit both the Drude-Lorentz and electron kinetic models to the experimental measurements. In the electron kinetic model, five fitting parameters are used: the electron injection rise time (τ_0), the shallow trap occupation time (τ_{Sh}), the deep trap occupation time (τ_{De}) and the weights of the two trap states (s and d). Thus the intention is to fit the function $f(t;\tau_0,\tau_{Sh},\tau_{De},s,d) = f(t,\boldsymbol{\beta})$ to the experimental data $y(t)$ by varying the parameters $\boldsymbol{\beta} = (\tau_0,\tau_{Sh},\tau_{De},s,d)$ minimizing the following first-order expression:

$$S(\boldsymbol{\beta}+\boldsymbol{\delta}) = \sum_{i}^{m} \left(y(t_i) - f(t_i;\boldsymbol{\beta}) - \nabla_\beta[f(t_i;\boldsymbol{\beta})]\boldsymbol{\delta}\right)^2$$

To evaluate the quality of the fit, the adjusted R^2 value is used to account for the number of fitting parameters (p) used. This is done to prevent overfitting, as the adjusted R^2 value only increases when including an additional parameter if it leads to a better fit than what is expected by random chance. [4]

$$R_{Adj}^2 = \frac{(1-R^2)(m-1)}{m-p-1}$$

Where R^2 is calculated using the standard formula:

$$R^2 \equiv 1 - \frac{\sum_i (y_i - f_i)^2}{\sum_i (y_i - \bar{y})^2}$$

The uncertainty on the fitted parameter is assumed to be one standard deviation on either side of the fitted value, giving a relatively high certainty (68.2%) that the true value is within the range stated.

[4] Theil, Henri. Economic Forecasts and Policy. Holland, Amsterdam: North (1961).

S3 Ab Initio Calculations of Hot Carrier Thermalization in Titanium Nitride

As the thermal electrons heat via electron-electron interactions, there are more collisions between the thermal electrons and the lattice vibrations. This is associated with a concurrent heating of the lattice and cooling of the electron system via the electron-phonon scattering events. The coupling between electrons and phonons can be described by an electron temperature-dependent coupling parameter G. Lin et al. [6] derived the following expression for an arbitrary density of electronic states, $\rho(\epsilon)$ and electron distribution function $f(E,T_e)$ as above:

$$G(T_e) = -\frac{\pi k_B \lambda\langle\omega^2\rangle}{\hbar\rho(E_F)} \int \rho^2(\epsilon) \frac{\partial f(E,T_E)}{\partial E} \partial E$$

with $\lambda\langle\omega^2\rangle$ being the electron-phonon mass enhancement parameter at the second moment of the phonon spectrum [6]. Neglecting the interaction between the nonthermal electrons and the phonons as the heating of the lattice is negligible in the short lifetime of the energetic electrons, we perform density-functional theory (DFT) calculations to determine the dependence of the electron-phonon coupling strength on the electron temperature in stoichiometric titanium nitride. Figure S3-1a shows the calculated band structure density of states and the associated temperature-dependent electron-phonon coupling parameter, G, is shown in Figure S3-2a. To

characterize the corresponding changes in temperatures, it is imperative to understand the electron and lattice heat capacities defined by:

$$C_e(T_e) = \int_{-\infty}^{\infty} dE\, \rho(E) E \frac{\partial f(E,T_e)}{\partial T_e} \qquad C_l(T_l) = \int_{0}^{\infty} dE\, P(E) E \frac{\partial n(E,T_l)}{\partial T_l}$$

where $P(E)$ is the phonon density of states and $n(E,T_l)$ the Bose occupation factor for the phonon system. Figures S3-1b and S3-1c show the temperature dependences of the electronic and lattice heat capacities respectively. We then estimate the electron-phonon lifetime using the expression:

$$\tau_{e-p} \approx \frac{1}{G}\left(\frac{C_e C_l}{C_e + C_l}\right)$$

and plot the electron-temperature dependence in Figure S3-2b shown to be under 200 fs even at higher temperatures.

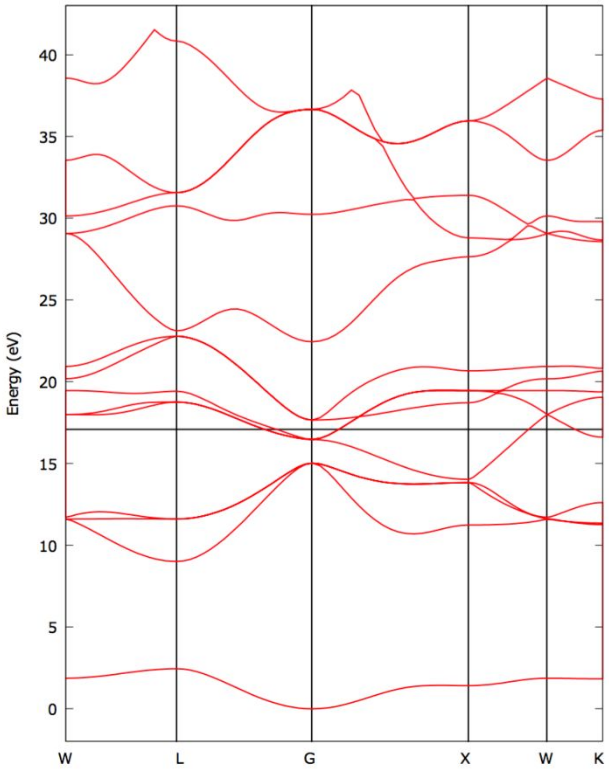

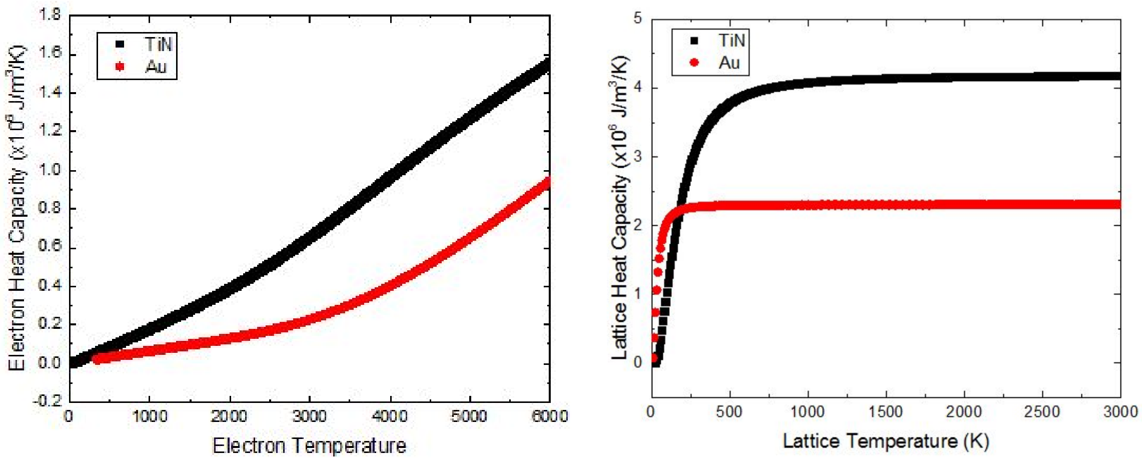

***Figure S3-1 | Density functional theory (DFT) calculations of electronic and thermal properties of TiN and Au*. *a**, electronic band structure and density of states of titanium nitride whose conductive properties are associated with the intersection of d-band states with the Fermi level. **b,** electronic heat capacity versus electron temperature showing a two linear regions with slightly different slopes. **c,** lattice heat capacity versus lattice temperature showing an increase even above 500K.*

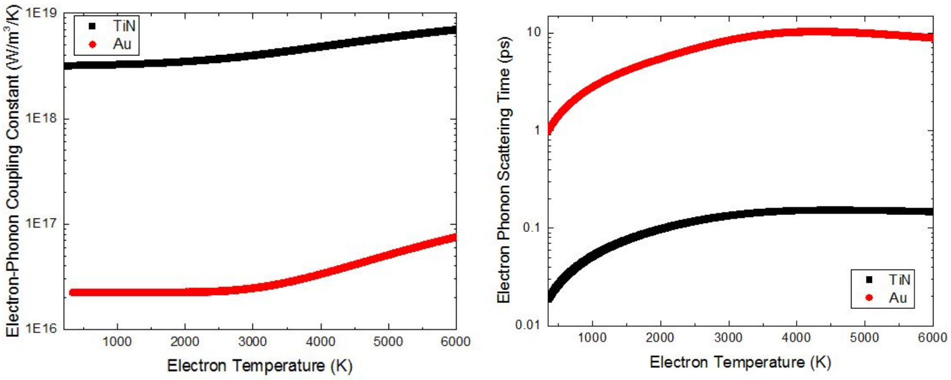

***Figure S3-2 | Calculated electron-phonon coupling and scattering time of TiN. a**, electron-phonon coupling constant of titanium nitride as a function of electron temperature displaying an increase over the entire range. **b,** electron-phonon scattering time as a function of electron temperature calculated from the parameters above.*

*[6] Brown, A. M., Sundararaman, R., Narang, P., Goddard, W. A. & Atwater, H. A. Ab initio phonon coupling and optical response of hot electrons in plasmonic metals. Phys. Rev. B **94,** 1–10 (2016).*

S4 Pump-Power Invariance of Trapping Lifetimes

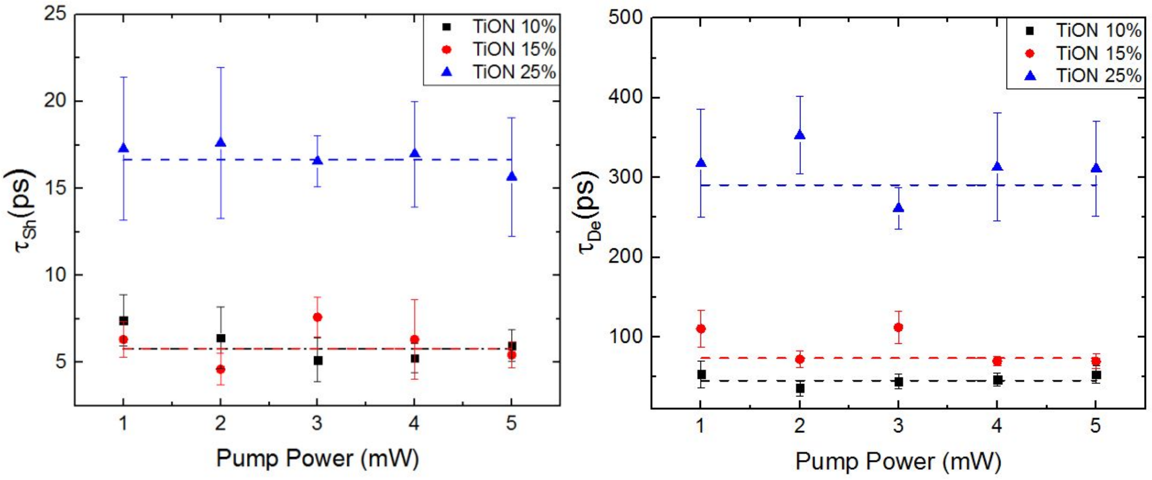

Figure S4 | Shallow and deep trapping lifetimes extracted at different pump powers. *The clear invariance of the lifetimes with pump power suggests that these are material properties and not hot-electron lifetimes as expected for trapping lifetimes.*

S5 Substrate Dependence of Electron Harvesting

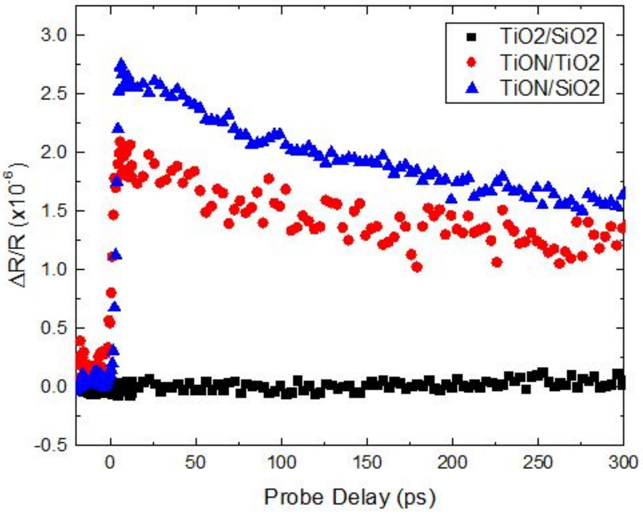

Figure S5 | Pump Probe Differential Reflectivity Measurements of different TiON Systems. *TiO_2/SiO_2, $TiON/TiO_2$ and TiO_2/SiO_2 interfaces measured with a 5 mW, 850 nm pump pulse and 150 µW, 1150 nm probe. The role of TiO_2 absorption in the differential reflectivity response is shown to be negligible, which is expected when exciting below the bandgap.*

S6 Sputtering Time versus Oxygen Content

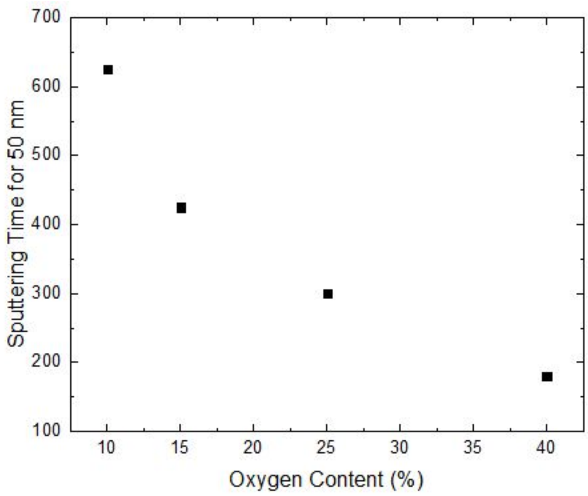

Figure S6 | Sputtering time for the various TiON films. *The increasing oxygen content in the film decreases the hardness and the films resistance to sputtering. As such there is a monotonic decrease of sputtering time for 50 nm of film. Note: as TiON 40% is 100nm, the sputtering time for 50 is a calculated value.*

S7 Band Diagrams

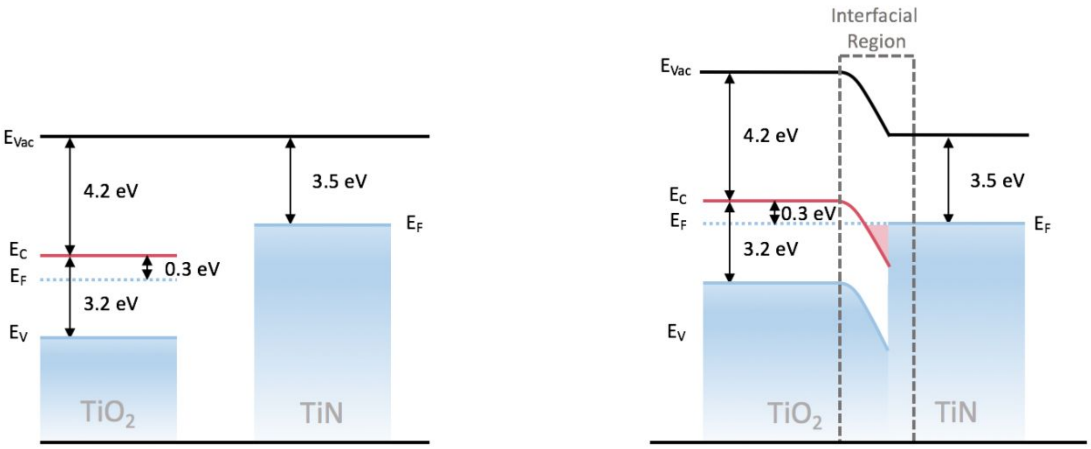

Figure S7 | Electronic band diagrams of TiO_2 and TiN. *(Left) Individual band diagrams of TiO_2 and TiN with respect to the vacuum energy level, E_{vac}. (Right) Theoretical band diagram of the contacted TiO_2 and TiN. As the TiN work function is smaller, electrons move into the TiO_2 at the interface resulting in downward bending of the conduction band.*
